# Cyclic decomposition explains a photosynthetic down regulation for *Chlamydomonas reinhardtii*

**DOI:** 10.1016/j.biosystems.2017.09.014

**Published:** 2017-12

**Authors:** Stephen P. Chapman, Marcelo Trindade dos Santos, Giles N. Johnson, Mauricio Vieira Kritz, Jean-Marc Schwartz

**Affiliations:** a Faculty of Biology, Medicine and Health, University of Manchester, Oxford Road, Manchester, M13 9PT, UK; b Laboratório Nacional de Computação Científica, Petrópolis, RJ, 25651-075, Brazil; c School of Earth and Environmental Sciences, University of Manchester, Oxford Road, Manchester, M13 9PT, UK; d Current address: Institute of Biological, Environmental and Rural Sciences, Aberystwyth University, Gogerrddan, Aberystwyth, SY23 3EE, UK

**Keywords:** Glycolysis, Mixotrophic growth, Metabolic model, Photosynthesis, Green algae, Flux balance analysis, Acetate metabolism, Cyclic electron flow

## Abstract

The regulation of metabolic networks has been shown to be distributed and shared through the action of metabolic cycles. Biochemical cycles play important roles in maintaining flux and substrate availability for multiple pathways to supply cellular energy and contribute to dynamic stability. By understanding the cyclic and acyclic flows of matter through a network, we are closer to understanding how complex dynamic systems distribute flux along interconnected pathways. In this work, we have applied a cycle decomposition algorithm to a genome-scale metabolic model of *Chlamydomonas reinhardtii* to analyse how acetate supply affects the distribution of fluxes that sustain cellular activity. We examined the role of metabolic cycles which explain the down regulation of photosynthesis that is observed when cells are grown in the presence of acetate. Our results suggest that acetate modulates changes in global metabolism, with the pentose phosphate pathway, the Calvin-Benson cycle and mitochondrial respiration activity being affected whilst reducing photosynthesis. These results show how the decomposition of metabolic flux into cyclic and acyclic components helps to understand the impact of metabolic cycling on organismal behaviour at the genome scale.

## 1 Introduction

It has been widely documented that cyclic structures play a central role in the homeostasis of biological systems (Qian and Beard, 2006, Cornish-Bowden and Cárdenas, 2008). For example, the tricarboxylic acid (TCA) cycle, also known as the Krebs cycle, is one of the most fundamental cycles for many species. The TCA cycle can be thought of as starting with the reaction of acetyl-CoA with oxaloacetate (OAA) producing citrate. With every turn of the cycle, the substrate OAA, a 4-carbon compound, is recycled and can therefore be used again for subsequent cycles. As with the TCA cycle, cycles play a pivotal role of retaining substrate matter and maintaining flux availability for multiple pathways to supply the cell with energy. In addition, cycles help maintain the organisational characteristics of a system, contributing to dynamic stability (Reznik and Segrè, 2010).

There are several ways to define “cycles” in networks or graphs, depending on the representation used. In metabolic network analysis, “substrate cycles” are generally defined as combinations of catabolic and anabolic reactions that interconvert one or more substrates into each other, resulting in no net production of substrate but potentially in the conversion of cofactors (Leiser and Blum, 1987). They can be enumerated in metabolic networks by techniques based on stoichiometric representations such as elementary mode and extreme pathways analysis (Schuster et al., 2000, Price et al., 2002, Gebauer et al., 2012). Kritz et al. (2010) introduced a way of analysing the cycling of matter in metabolic networks by adapting a pre-existing algorithm used to inspect cycles in ecological food webs (Ulanowicz, 1983). The adapted algorithm represents the metabolic network as a bipartite graph of metabolites and reactions; it first enumerates all graph-theoretical cycles in the network, then uses flux values associated to arcs to iteratively extract the cycles from the graph, leaving a residual acyclic graph in the case of an open network. Kritz et al. (2010) were able to observe the metabolic changes that occur with gene knockout experiments, unveiling novel hypotheses on how organismal growth can be optimised.

*Chlamydomonas reinhardtii* is a unicellular green algae which has been a model organism for the study of photosynthesis and cellular function for the past 50 years (Harris, 2001). Recent concerns over fossil fuel depletion have led to research into the use of micro-algae such as *C. reinhardtii* to produce sustainable biofuels (Hannon et al., 2010). The cells of this alga fix carbon from the atmosphere via the Calvin-Benson cycle, the products of which can be channelled into tri-acyl glyceride lipid bodies, providing the building blocks for biofuel (Merchant et al., 2012). The high energy requirement of this process is met by light capture in the photosynthetic reactions. Photosynthesis is a highly tuned system and is one of the most regulated processes in nature. To improve biofuel yields from micro-algae, we need to fully understand the connections between photosynthesis and metabolism.

Here, we apply the abovementioned cycle decomposition methodology to investigate the cyclic flux distribution in *C. reinhardtii*, using a published and validated genome-scale metabolic model (GEM) of the organism, iRC1080 (Chang et al., 2011), in an attempt to present a comprehensive description of cyclic and acyclic metabolic fluxes for the organism. Using this methodology, we derive novel hypotheses that explain the down-regulation of photosynthesis, which has been widely observed when cells experience a change from phototrophic to mixotrophic growth (Johnson and Alric, 2013, Johnson et al., 2014). We show that acetate addition results in the modulation of key glycolytic and mitochondrial reactions, tipping the requirement of reducing power and ATP that feeds back on the photosynthetic reactions.

## 2 Methods

The procedure used for the decomposition of metabolic fluxes into cyclic and acyclic components consists of two distinct steps. The first is the extraction of a mass-consistent subset of the iRC1080 model, whilst the second is the enumeration of cycles and decomposition of flux.

The metabolic network is represented by a directed bipartite graph in which metabolites and reactions are represented by distinct nodes, belonging to two disjoint sets that are connected by arcs between nodes of each set. An arc from a metabolite node to a reaction node indicates that the metabolite is a substrate for that reaction. In contrast, an arc from a reaction node to a metabolite node indicates that the metabolite is a product of that reaction. Each reaction may therefore have one or multiple associated substrates and products, and each metabolite may be associated with multiple reactions. The bipartite graph representation contains the same information about the metabolic network as a hypergraph (Klamt et al., 2009). This is similar to the representation used by Petri nets, which have been frequently applied to metabolic networks (Koch et al., 2005, Rohr et al., 2010, Zevedei-Oancea and Schuster, 2011).

A mass flux is then associated to each arc of the bipartite graph. The mass flux takes into account the mass, stoichiometric coefficient and molar fluxes (e.g. obtained from Flux Balance Analysis) for each species involved in the reaction. The main reason for using mass fluxes rather than molar fluxes is that the cycle decomposition algorithm requires quantities that are conserved at nodes, whereas molar fluxes are not conserved. For example, when fructose 1,6-biphosphate is broken down into glyceraldehyde 3-phosphate and dihydroxyacetone phosphate, 1 mol is transformed into 2 mol, thus the number of moles is not conserved; nevertheless the sum of masses of glyceraldehyde 3-phosphate and dihydroxyacetone phosphate is equal to the mass of fructose 1,6-biphosphate.

In order to assign a mass flux to every arc in the metabolic network, certain reactions had to be modified in the representation. Generic compounds such as glycogen and starch are composed of a core structure associated with a varying number of branched repeats, hence it is impossible to attribute a definite mass value for such generic compounds – they can have distinct masses in distinct occurrences. In addition to generic compounds, proteins and protein complexes described in biochemical reactions can create apparent mass imbalances, despite not being part of the metabolic network. Similarly, transfer (tRNA)-amino-acid complexes, present in GEMs, can introduce apparent mass inconsistencies. In addition, smaller imbalances can appear when the chemical description of substrates and products are associated with different pH levels, resulting in an inconsistency in the number of hydrogen atoms.

Mass imbalances originating from the apparent imbalances described above were solved locally, i.e. by correcting the mass of the species to eliminate the apparent mass imbalance, as described in Kritz et al. (2010). Moreover, the most ubiquitous cofactors were left out from our representation and replaced by gateways (Supplementary file 1, Table S1), except in the thykaloid lumen where reactions were left unchanged. Gateways are model artefacts and can be understood as channels for exchanging matter with the environment. The presence of cofactors has the potential to create spurious network connections, as cofactors are involved in multiple reactions that are not necessarily co-located in space and time. In each occurrence of a cofactor molecule (water, for instance) in the model, it was replaced by a gateway specific to the corresponding reaction. This procedure eliminates the spurious connections between the water species and all reactions in which it appears.

Parsimonious Flux Balance Analysis (pFBA) was used to calculate the molar flux in each reaction, which was then converted into a mass flux. pFBA finds a flux distribution that optimises the objective function and at the same time minimises the total sum of fluxes (Lewis et al., 2010). This implies that reactions that aren’t needed to contribute to the optimal objective have a flux of zero, and that the flux in internal loops is minimised. This technique is widely used to avoid the degeneracy of standard FBA solutions and was shown to offer highly reliable results when compared to experimentally determined fluxes (Machado and Herrgård, 2014). pFBA was performed using the COBRA toolbox (Becker et al., 2007) in conditions of phototrophic growth (growth using only light as an energy source) and mixotrophic growth (growth using both light and acetate, an organic carbon source). To ensure the set of fluxes for each condition were representative of physiological traits, further constraints were applied to mimic experimental conditions with regards to biomass, known carbon metabolism pathways and experimentally determined photosynthetic rates. The scripts used to calculate the flux distributions were published in the supplementary material of Chapman et al. (2015), where the exact constraints applied to mimic experimental conditions can be found.

Tarjan’s algorithm (Tarjan, 1973) was used to enumerate all cycles in the bipartite graph. The algorithm seeks to enumerate cycles by traversing the network along an elementary path. An elementary path can be described as a path in which there is no repetition of any node. Following identification, the cycle is enumerated and the algorithm continues by backtracking to the previous node, following another arc to traverse. The process is repeated until all cycles have been enumerated. Tarjan’s algorithm provides the most suitable method for cycle enumeration with regards to computational efficiency, due to pruning methods that avoid searching in already traversed paths that would return no new cycles, reducing the search space and increasing algorithm efficiency. Antiporter reactions were split into two separate transport reactions before running Tarjan’s algorithm in order to prevent connections between compounds located in the same compartment where no actual transfer of mass occurs (Supplementary file 1, Table S2).

The final phase was the cyclic decomposition of the flux. The complete algorithm was described in Kritz et al. (2010) and is here summarised briefly. First, a critical arc is identified in the network, defined as the arc bearing a minimal mass flux among all cycles. The set of all cycles sharing this critical arc is then found and defined as its nexus. Next, the probability of mass flux entering any of the nexus cycles via any node adjacent to the critical arc is calculated, and the mass flux is distributed among the nexus cycles in proportion to their respective circuit probability. Then each cycle belonging to the nexus is removed by subtracting its flux from the network. The algorithm then reiterates by searching for the next critical arc and repeats the process until the resulting network is void of cycles.

## 3 Results

### 3.1 Correction of mass inconsistencies

The metabolic model *i*RC1080 representing the model organism *C. reinhardtii* was used for this work (Chang et al., 2011). The molecular mass of each compound was calculated from their chemical formula published in the supplementary material of the original *i*RC1080 paper (Chang et al., 2011). Some species are attached to side chains (represented by an ‘R' in the formula) of unknown masses, allowing us to adjust, if needed, the overall species mass, to balance a specific reaction.

Some specific examples of correction methodology are described below.

Example 1 Mass inconsistency due to an unknown protein mass.

Consider R_CEF: this reaction is an important photosynthetic regulatory mechanism in which electrons are re-directed about photosystem I, resulting in increased ATP production.

R_CEF: fdxrd[u] + (2) h[h] + (2) pccu2p[u] −> fdxox[u] + (4) h[u] + (2) pccu1p[u]

This reaction was imbalanced by 412.56 g/mol, attributed to a substrate. Upon closer inspection of the reaction, it was apparent that one of the substrates, plastocyanin (pccu2p[u]), a protein present in the reactions of photosynthesis, was without a formula and without a mass (while the mass of ferredoxin was properly accounted for). We have attributed to plastocyanin the mass necessary to balance the corresponding reaction.

Example 2 Mass inconsistency due to generic compounds.

Consider R_3HAD60: this reaction is 3-hydroxyacyl-[acyl-carrier-protein] dehydratase, located in the chloroplast catalysing the following fatty acid biosynthesis reaction.

R_3HAD60: 3hhexACP <= => thex2eACP + h2o

3hhexACP[h] is 3-Hydroxyhexanoyl-[acyl-carrier protein] (species formula = C6H11O2SR); thex2eACP is trans-Hex-2-enoyl-[acyl-carrier protein] (species formula = C6H9OSR).

Both formulae for the reactant and species contain an ‘R’ indicating a side chain that corresponds to an unknown number of repeating units associated to each residue molecule, therefore the mass of this generic species can not be accurately specified. We have attributed to generic compounds the specific mass values needed to balance the reactions in which they occur.

Example 3 Correction due to missing protons.

Consider R_CCP2m, a cytochrome c peroxidase mitochondrial reaction.

R_CCP2m: 2 M_focytc_m + M_h2o2_m <= => 2 M_ficytc_m + 2 M_h2o_m

where M_focytc_m is ferrocytochrome c, mass 908.82 g/mol; M_h2o2_m is hydrogen peroxide, mass 34.01 g/mol; M_ficytc_m is ferricytochrome c, mass 908.82 g/mol; M_h2o_m is water, mass 18.01 g/mol. We needed to add a small gateway to the left side, to achieve mass balance. This small gateway corresponds to the mass of two missing protons in the substrates. Small gateways, in general, are needed to adjust the masses of missing protons, due to reaction description in distinct protonation states.

### 3.2 Enumeration of cycles

We first applied parsimonious Flux Balance Analysis to the iRC1080 model in order to obtain the molar fluxes (mmol/gDW/h) under mixotrophic and phototrophic conditions. Then, we calculated mass fluxes (mg/gDW/h) and applied the decomposition algorithm to the non-zero flux sub-networks under these growth conditions. We have considered in our calculations only mass fluxes ≥ 0.0001 to filter out numerical noise. The first step of cycle decomposition corresponds to cycle enumeration, and we performed this analysis only to four sub-models of interest because of their importance in housing reactions belonging to central carbon metabolism: the thylakoid lumen, chloroplast, mitochondria, and a sub-model composed by reactions and substrates in all three. The restriction to sub-models is operationally important because the number of cycles become too large and not manageable for the complete network. The numbers of cycles obtained in these sub-models under the two growth conditions of interest are shown in Table 1 and the full lists of cycles are found in Supplementary files 2 and 3.

**Table 1:** Cycles resulting from cyclic decomposition for key compartments for phototrophic and mixotrophic growth, showing common and unique cycles to both conditions.

Compartment	Total phototrophic cycles	Total mixotrophic cycles	Common cycles to both conditions	Unique phototrophic cycles	Unique mixotrophic cycles
Mitochondrion	6	5	3	3	2
Thylakoid lumen	20	4	4	16	0
Chloroplast	587	1885	51	536	1834
Mitochondrion, thylakoid lumen and chloroplast	5151	1673	52	5099	1621

### 3.3 Cycle decomposition reveals acetate modulates changes in TCA flux and mitochondrial respiration

**Fig. 1:**
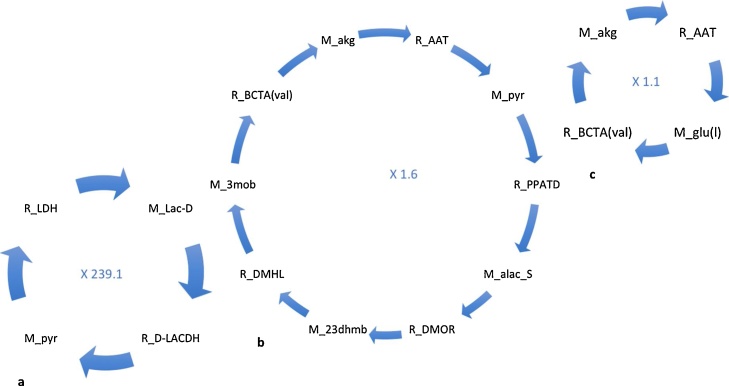
Cycles unique to phototrophic conditions in the mitochondrion: (a) pyruvate regeneration, (b) pyruvate recycling linked to 2-oxogluterate recycling and (c) glutamate and 2-oxogluterate cycling. Species starting with ‘R_’ indicate reactions whilst ‘M_’ indicate metabolites. Abbreviations: 23dhmb: 2,3-dihydroxy-3-methylbutanoate, 3mob: 3-methyl-2-oxobutanoate, AAT, alanine aminotransferase, alac_S: 2-acetolactate, BCTA(val): branched-chain-amino-acid transaminase (valine forming), akg: 2-oxoglutarate, D-LACDH: D-lactate dehydrogenase, DMHL: 2,3-dihydroxy-3-methylbutanoate hydro-lyase, DMOR: 2,3-dihydroxy-3-methylbutanoate NADP+ oxidoreductase (isomerizing), glu: glutamate, lac-D: lactate; LDH: lactate dehydrogenase; PPATD: pyruvate acetaldehydetransferase (decarboxylating); pyr: pyruvate.

The lists of cycles and their associated flux after decomposition following the algorithm of Kritz et al. (2010) algorithm are found in Supplementary files 4 and 5. We first consider cycles appearing in the mitochondrion due to its importance in metabolism. The mitochondrion is the location of the TCA cycle and the respiratory electron transport chain providing a major source of ATP to fuel metabolism. We observe 3 cycles in the mitochondrion being common to both phototrophic and mixotrophic conditions, 3 cycles unique to phototrophic growth and 2 unique mixotrophic cycles. The unique cycle attributable to phototrophic metabolism which carries the largest mass flux involve pyruvate and lactate metabolism (mass_flux 239) (Fig. 1a). The other two cycles unique to phototrophic conditions involve the cycling of glutamate and 2-oxoglutarate (mass_flux 1) (Fig. 1b) and a cycle involving pyruvate and 2-oxoglutarate metabolism (mass_flux 2) (Fig. 1c).

**Fig. 2:**
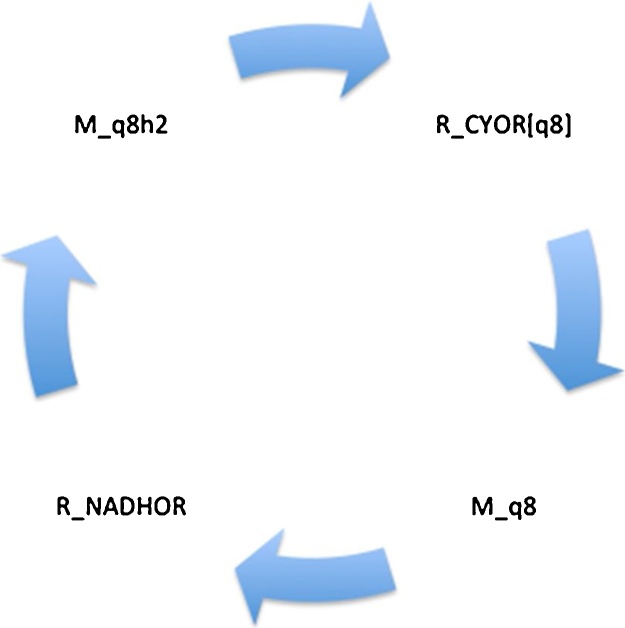
Mixotrophic cycle carrying the greatest mass flux within the mitochondrion. Species starting with ‘R_’ indicate reactions whilst those with ‘M_’ indicate metabolites. Abbreviations: q8: ubiquinone; q8h2: ubiquinol; NADHOR: ubiquinone oxidoreductase complex I; CYOR: ubiquinol-cytochrome c oxidoreductase complex III.

In mixotrophic conditions the unique cycle carrying the greatest mass flux involves the mitochondrial respiratory electron transport chain (mass_flux 1244). This cycle involves the continued recycling of ubiquinone (M_q8) and ubiquinol (M_q8h2), the two key components involved in facilitating the electron transport between protein complexes, to generate ATP (Fig. 2). Associated to this cycle are the reactions of ubiquinone oxidoreductase Complex I (R_NADHOR) and ubiquinol-cytochrome c oxidoreductase Complex III (R_CYOR(q8)). This quinone pool accepts and donates electrons and protons (H+) to appropriate acceptor molecules. Coupled to the flux of electrons, protons are pumped out of the mitochondrion, resulting in the generation of a proton-motive force to drive ATP synthesis (Chaban et al., 2014).

Inspections of the residual acyclic graphs for mixotrophic growth suggest that acetate is directly transported into the mitochondrion, and ultimately branched towards the reactions of gluconeogenesis replacing the need of pyruvate. In this scenario, pyruvate was channelled into other pathways such as valine, formate and fatty acid metabolism (Supplementary file 1, Table S3).

The unveiled cycles can feed-forward to enhance other cycles downstream in metabolism. Phototrophic cycles have shown that increased flux through pyruvate metabolism and its entry into the TCA cycle allows for the production of TCA cycle intermediates in excess, which was otherwise redundant in the mixotrophic case. Instead, cycling components of the mitochondrial electron transport chain, which are active under mixotrophic growth, are otherwise redundant in phototrophic metabolism and it is only with cyclic decomposition that these perturbations can be analysed.

### 3.4 Cycles within the thylakoid lumen reveal an increased cycling of ascorbate and increased flux carried through the photosystem II reaction associated with phototrophic growth

**Fig. 3:**
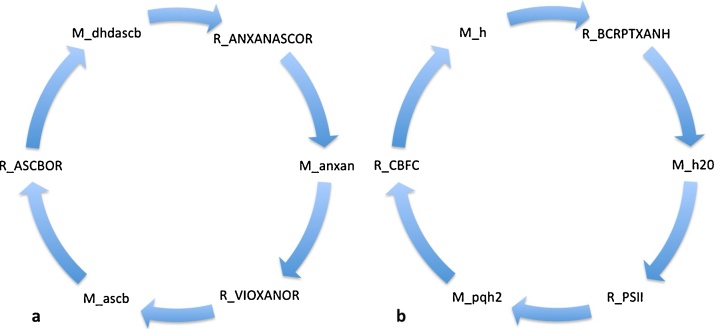
Phototrophic cycle decomposition revealed two prominent cycles taking place within the thylakoid lumen: (a) ascorbate metabolism and (b) recycling of plastoquinone associated with proton production (M_h) and activation of photosystem II (R_PSII), responsible for oxygen evolution. Species starting with ‘R_’ indicate reactions whilst ‘M_’ indicate metabolites. Abbreviations: anxan: antheraxanthin; ANXANASCOR: ascorbate oxidoreductase; ascb: ascorbate; ASCBOR: ascorbate:oxygen oxidoreductase; BCRPTXANH: beta-cryptoxanthin hydroxylase; CBFC: cytochrome b6/f complex; h: proton; dhdascb: dehydroascorbate; pqh2: reduced plastoquinone; VIOXANOR: violaxanthin: ascorbate oxidoreductase.

Our next analysis considered the cycles within the thylakoid lumen. This sub-compartment of the chloroplast houses the light driven electron transport reactions of photosynthesis. We found no unique cycle associated with the mixotrophic condition but 16 cycles were unique to phototrophic growth and 4 cycles were common to both conditions. The highest scoring unique phototrophic cycle (mass_flux 0.019) involved ascorbate metabolism, consisting of the cycling of ascorbate (M_ascb) by the violaxanthin:ascorbate reductase reaction (R_VIOXANOR), resulting in the re-oxidation of ascorbate (M_dhdascb) (Fig. 3a). This cycle describes part of the xanthophyll cycle, a well-known photo-protective cycle for plants and green algae to adapt and maintain efficient photosynthesis under fluctuating light conditions (Jahns and Holzwarth, 2012). In addition, ascorbate was involved in 9 other cycles carrying a much lower mass flux of 0.002. The remaining 6 cycles all involved components of the photosynthetic electron transport chain associated with carotenoid biosynthesis with the highest mass flux being 0.0002 as seen in Fig. 3b.

The 4 cycles common to both conditions all involved cycling of matter through the reactions of photosynthetic electron transport involving both photosystems I and II. This suggests the importance of *both* photosystems to support both phototrophic and mixotrophic biomass. Furthermore, the finding that flux through part of the xanthophyll cycle was observed with phototrophic simulations is a proof of principle that cyclic decomposition can be used to unveil important biological cycles otherwise ignored by stoichiometric analysis alone.

### 3.5 Acetate metabolism results in activation of the oxidative pentose phosphate pathway rather than glycolysis

To gain a further understanding of cycles across compartments, we expanded the analysis to include the mitochondria, thylakoid lumen and chloroplast compartments together. The addition of the chloroplast, the largest organelle in *C. reinhardtii*, resulted in a vast increase in the number of unique cycles for both phototrophic and mixotrophic conditions (5099 and 1621 cycles respectively). Only 52 cycles were found to be common to both growth regimes.

**Fig. 4:**
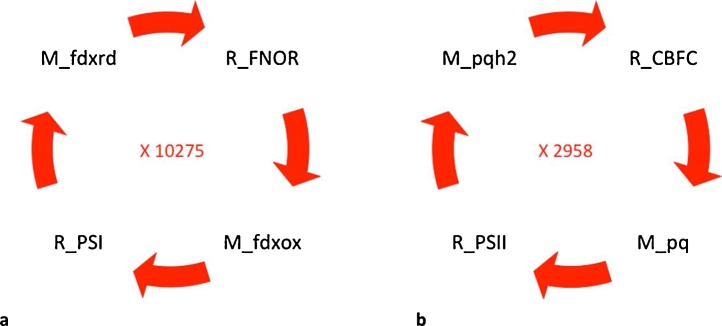
Cycles common to both mixotrophic and phototrophic growth conditions that carried the highest mass flux for each condition when the mitochondria, thylakoid lumen and chloroplast compartments were analysed together. The number displayed within each cycle represents the mass flux obtained for each condition. Species starting with ‘R_’ indicate reactions whilst ‘M_’ indicate metabolites. Abbreviations: CBFC: cytochrome b6/f complex; fdxox: oxidized ferredoxin; fdxrd: reduced ferredoxin; FNOR: ferredoxin-NADP+ reductase; PSI: photosystem I; PSII: photosystem II; pq: oxidized plastoquinone.

For both conditions, the cycle carrying the greatest mass flux included the Calvin-Benson cycle (Fig. 4a) associated with different glycolytic reactions. The occurrence of the Calvin-Benson cycle intermediates (D-ribulose 1,5-bisphosphate, ribulose 5-phosphate) was used as an indicator of the importance of the Calvin-Benson cycle for each growth regime. Out of these common cycles for phototrophic and mixotrophic conditions, the cycles carrying the greatest mass fluxes (10275 and 2958) both involved important photosynthetic complexes (Fig. 4).

**Fig. 5:**
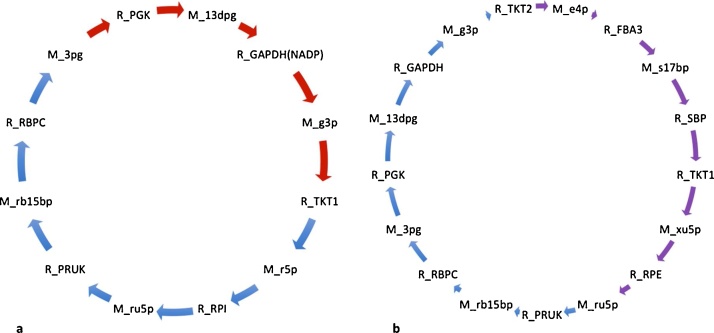
Unique cycles resulting from the mitochondrion, thylakoid lumen and chloroplast. (a) Phototrophic cycles included reactions of the Calvin-Benson cycle (blue arrows) and glycolysis (red arrows) whilst (b), mixotrophic growth resulted in the same Calvin-Benson cycle reactions (blue arrows) associated with the reactions of the pentose phosphate pathway (purple arrows). Species starting with ‘R_’ indicate reactions whilst ‘M_’ denotes metabolites. Abbreviations: 13dpg: 3-phospho-d-glyceroyl phosphate; 3 pg: 3-phospho glycerate; e4p: erythrose 4-phosphate; FBA3: sedoheptulose 1,7-bisphosphate D-glyceraldehyde-3 phosphate lyase; g3p: glyceraldehyde 3-phosphate; GAPDH: glyceraldehyde 3 phosphate dehydrogenase; GAPDH(NADP): glyceraldehyde 3 phosphate dehydrogenase (NADP+); PGK: phosphoglycerate kinase; PRUK: phosphoribulokinase; r5p: ribose 5-phosphate; RBPC: ribulose-bisphosphate carboxylase; rb15 bp: ribulose 1,5-bisphosphate; RPE: ribulose-5-phosphate 3-epimerase; RPI: ribose-5-phosphate isomerase; ru5p: ribulose 5-phosphate; s7p: sedoheptulose 1,7-bisphosphate; s17 bp: sedoheptulose 1,7-bisphosphate; TKT1: transketolase 1; TKT2: transketolase 2; xu5p: xylulose 5-phosphate. (For interpretation of the references to colour in this figure legend, the reader is referred to the web version of this article.)

The occurrence of the Calvin-Benson cycle enzyme RuBisCo (ribulose bisphosphate carboxylase) was used as an indicator of the importance of the Calvin-Benson cycle for each growth regime. Out of the 5099 unique phototrophic cycles, 5079 cycles (99.6%) contained the classic Calvin-Benson cycle enzyme RuBisCo. The cycle to carry the largest mass flux (322) was also associated with RuBisCo combined with reactions of gluconeogenesis (Fig. 5a).

There were 1062 unique mixotrophic cycles that contained RuBisCo, or 20.8% of the unique mixotrophic cycles, suggesting a reduced importance of a fully operational Calvin-Benson cycle under mixotrophic metabolism. It is apparent that cells undergoing mixotrophic growth on acetate and CO2 do exhibit a preference for acetate metabolism over CO2 sequestration, but do utilise both carbon sources nevertheless. The fact that we were able to observe ∼20% cycles involving RuBisCo is of much interest, since FBA predicted inactivity of this enzyme under mixotrophic conditions (Chapman et al., 2015), even though carbon fixation by RuBisCo is seen experimentally in mixotrophic conditions (Johnson et al., 2014). This finding highlights the added value of cyclic decomposition following FBA.

The cycle to carry the greatest mass flux for mixotrophic metabolism, when the mitochondria, thylakoid lumen and chloroplast compartment were considered, consisted of the cycling of oxaloacetate, with the involvement with a membrane transporter that transports oxaloacetate between the cytosol and mitochondria, in exchange for malate (mass flux 354). Mixotrophic analysis suggested a prominent occurrence of the oxidative pentose phosphate (OPPP) cycle in carbon metabolism. The OPPP results in the synthesis of carbon skeletons for nucleotide synthesis and provides a source of reductant in the form of NAPDH for fatty-acid synthesis. Sedoheptulose 7-phosphate (S7P) (M_s7p, Fig. 5b) is the key metabolite used for nucleotide biosynthesis by the reductive steps of the PPP and sugar re-entry into the Calvin-Benson cycle (Kruger and Von Schaewen, 2003) (Fig. 4b). As seen in Table 2, the proportion of cycles containing S7P was greater for mixotrophic growth, 61% (987 cycles), than for phototrophic growth, 37% (1894 cycles).

**Table 2:** Percentage of cycles containing key metabolites of the pentose phosphate pathway and glycolysis for phototrophic and mixotrophic growth.

Pathway	Phototrophic (% unique cycles)	Mixotrophic (% unique cycles)
Pentose phosphate pathway	36.9	58.3
Glycolysis	49.8	34.8

When Fig. 5(a) and (b) are compared, the presence of metabolites resulting from glycolysis only appears in the cycle carrying the highest mass flux for phototrophic growth, suggesting an important role of glycolysis in mixotrophic metabolism. To further investigate the functioning of glycolysis associated with each growth condition, we looked at the reaction R_GAPDH(nadp), which is responsible for the production of triose phosphate and NADP from 3-phospho-d-glyceroyl in the chloroplast, as an indicator of glycolytic activity. Occurrence of this key glycolytic node shows a 43.1% increase associated with phototrophic metabolism (Table 2). These results suggest that acetate has the effect of increasing OPPP activity, whilst decreasing reactions associated with the glycolytic pathway.

**Fig. 6:**
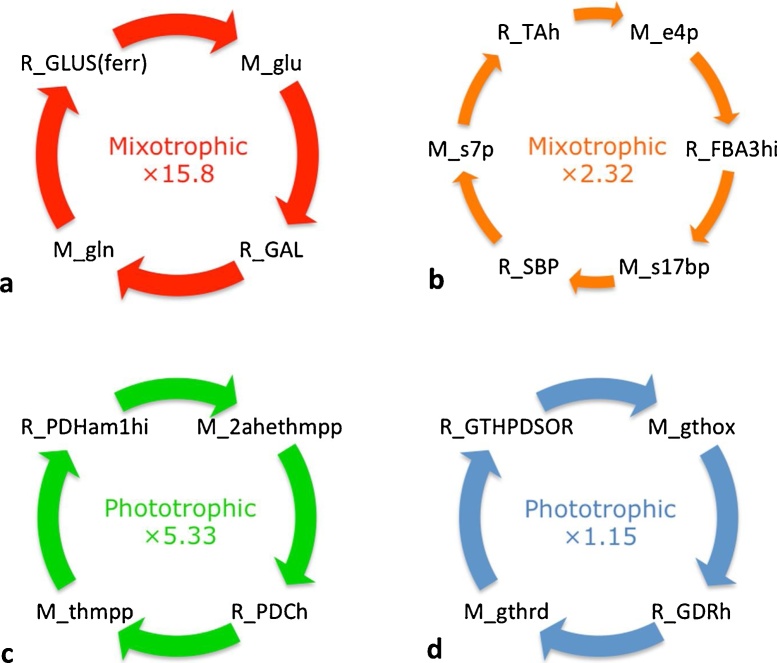
Common cycles within the mitochondrion, thylakoid lumen and chloroplast carrying the largest mass flux. Numbers indicate the fold change of mixotrophic vs phototrophic flux in a-b, phototrophic vs mixotrophic flux in c-d. (a) Mixotrophic growth results in increased cycling of glutamine and galactose and (b) increased pentose phosphate pathway activity. (c) Phototrophic growth resulted in an increase of pyruvate metabolism within the mitochondrion and (d) increased cycling of glutathione. Species starting with ‘R_’ indicate reactions whilst ‘M_’ denotes metabolites. Abbreviations: 2ahethmpp: 2-(alpha-hydroxyethyl)thiamine diphosphate; e4p: erythrose 4-phosphate; FBA3hi: sedoheptulose 1,7-bisphosphate D-glyceraldehyde-3-phosphate-lyase; GAL: glutamate-ammonia ligase; GDR: glutathione-disulfide reductase; gln: glutamine; gthox: oxidized glutathione; gthrd: reduced glutathione; GTHPDSOR: glutathione:protein-disulfide oxidoreductase; glu: glutamate; GLUS(ferr): glutamate synthase (ferredoxin dependent); PDCh: pyruvate decarboxylase; PDHam1hi: pyruvate dehydrogenase; s17 bp: sedoheptulose 1,7-bisphosphate; s7p: sedoheptulose 7-phosphate; SBP: sedoheptulose-bisphosphatase; TAh: transaldolase; thmpp: thiamine diphosphate.

Out of the 52 cycles common to both growth conditions, there were 4 cycles with large mass flux differences between the two conditions. Fig. 6(a) and (b) displays cycles that increased as a result of mixotrophic growth whilst Fig. 6(c) and (d) displays cycles that increased with phototrophic growth. Cycles with the largest absolute difference with respect to mixotrophic metabolism involve glutamate (M_glu) and galactose synthesis (m_GAL) 1500% increase), and carbon assimilation into the OPPP to produce the intermediate sugars erythrose 4-phosphate (M_e4p), sedoheptulose 7-phosphate (M_s7p) and sedoheptulose 1,7-bisphosphate (M_s17 bp) (132% increase).

Phototrophic metabolism resulted in increasing matter being cycled through reactions involving pyruvate metabolism within the mitochondrion and an increased shuttling of malate out of the mitochondria antiporter (R_MALOAATm) synchronised with the import of oxaloacetate, representing a 433% and 15% increase respectively. These results collectively suggest that both phototrophic and mixotrophic growth are able to channel photosynthate towards the OPPP and to metabolise pyruvate into alternative pathways as and when needed.

## 4 Discussion

The main objective of this research was to investigate metabolic cycles occurring as a result of altering the growth conditions of the model organism *C. reinhardtii*. The decomposition of metabolic flux into cyclic and acyclic components, as introduced by Kritz et al. (2010), gives a finer-grained view of the cycles of metabolic matter than more traditional stoichiometric analysis methods. We were able to describe the interlocking of different cycles and how their structure and activity is affected by changes in the growth environment. We showed that the presence of acetate in the growth medium results in a fundamental change in the primary routes of carbon metabolism, which feeds back into the reactions of photosynthesis.

To understand the effect of acetate assimilation on overall metabolism, it is first important to consider the cycles unveiled in phototrophic conditions. Phototrophic cultures fix CO_2_ into triose phosphate. Triose phosphate is incorporated into metabolism by the reactions of gluconeogenesis to generate hexose sugars, which can be stored as starch reserves. In times of need, these reserves can be degraded by glycolytic pathways to release ATP. Glycolysis is compartmentalised in *C. reinhardtii* with the upper half occurring in the chloroplast and the lower half in the cytosol (Kruger and von Schaewen, 2003). Our analysis revealed a positive feedback loop commencing with the fixation of CO_2_ and culminating with an increased pyruvate cycling (Fig. 1a) and oxoglutarate cycling (Fig. 1b and c). The role of oxogluterate is well characterised in the literature, and known for its essential role of incorporating ammonia into amino acids in the chloroplast, driven by photosynthesis (Paul and Foyer, 2001). The increased cycling of reduced and oxidized plastoquinone (Fig. 3b) ensures a constant traffic of electrons through the photosynthetic transport chain, specifically though PSII, the photosystem responsible for oxygen evolution. This cycle is therefore closely associated to the cycle observed in Fig. 4a: the products of photosynthesis, ATP and NADPH are utilised by the Calvin-Benson cycle for the fixing of CO_2_ and incorporation of photosynthate into gluconeogenesis. An increased flux of glycolysis would ensure a constant production of pyruvate, maintaining substrate material required for continued TCA activity. As a result, flux is seen to enhance further cycling of TCA intermediates, malate, citrate and oxaloacetate finally resulting in an increased flux of malate out of the mitochondria associated with an import of oxaloacetate to continue the TCA cycle, allowing the organism to utilise malate elsewhere.

For mixotrophic cultures, perturbations in the environmental conditions by the addition of acetate have a profound effect on metabolism, as unveiled by cyclic decomposition. We showed that mixotrophic growth results in an increased number of cycles involving the OPPP intermediates, ultimately enhancing further cycles involving mitochondrial respiration whilst reducing the flux associated with carbon fixation, reducing the requirement of NAPDH. The OPPP can be thought of as a carbon sink, channelling photosynthate into nucleotide synthesis, which explains associated increases in both growth rate and biomass observed with a mixotrophic growth regime. The PPP occurs in two steps: a reductive and an oxidative step. The oxidative PPP results in the production of NAPDH, without any net gain of ATP (Kruger and von Schaewen, 2003). NAPDH is an electron donor that provides a source of electrons in the mitochondrion to sustain electron transfer during respiration (Sandmann and Malkin, 1983). The continual cycling of reduced and oxidized ubiquinone ensures a faster rate of electron acceptance and proton migration to prevent any toxic accumulation of NADPH. Here we see that activation of the OPPP occurs as carbon fixation into the Calvin-Benson cycle is reduced, explaining a down-regulation of photosynthesis, and furthermore initiates a tight link with the mitochondrial respiratory electron chain. Fig. 6b suggests the OPPP is not a unique attribute associated with mixotrophic growth, as phototrophic simulations also reveal the channelling of material into these pathways, but at reduced rates.

An updated metabolic model of *C. reinhardtii* (*i*Cre1355) was recently published (Imam et al., 2015) which showed a marked improvement when predicting maximum TAG yields in comparison to *i*CR1080. With regards to growth rates, *i*Cre1355 showed no improvement over *i*CR1080 unless nitrogen starvation is considered. Here, *i*Cre1355 focused specifically on nitrogen starvation and changes of light regime on growth. As such, *i*Cre1355 represents a vast improvement over *i*CR1080 when nitrogen metabolism (depletion) and TAG accumulation are considered. Nevertheless the study we present follows on from previous work in which we are investigating why acetate represses photosynthesis, a very distinct question to one against which *i*Cre1355 was validated. Upon closer comparison of *i*CR1080 and *i*Cre1355, it appears the photosynthetic reactions of iCR1080 and *i*Cre1355 are identical and both include the CEF reaction. This piece of research follows on from published work made with iCR1080, and as such we noted and corrected multiple erroneous reactions associated with CEF and the movement of photons from the stroma to the lumen that occurs as a result of CEF (Chapman et al., 2015). Since we had already corrected for these key reactions, we decided it best to keep with *i*Cr1080, as we had already made these necessary adjustments with the reactions of photosynthesis and a true representation of these reactions is critical to our study on why acetate represses photosynthesis.

There are other possible representations of metabolic networks for the enumeration of feasible paths or cycles. Arita (2004) linked metabolic compounds by taking into account carbon atom paths, in order to avoid spurious connections between metabolites that did not share any atomic connections. The same property is achieved in our representation by taking into account the mass of compounds: this guarantees that only mass-conserving paths are allowed, therefore no connection is possible between compounds that do no share any atomic connection. Elementary Flux Modes (EFM) are another extremely useful representation for metabolic networks. Gebauer et al. (2012) demonstrated that it is possible to enumerate all substrate cycles in a genome-scale network using EFMs, but an additional algorithm is needed to quantify their flux level. We previously published an algorithm towards that aim (Schwartz and Kanehisa, 2006), however that algorithm did not scale up well in large networks and tended not to converge in networks containing cycles. The algorithm of Kritz et al. (2010), on which this current work is based, addresses both problems: it works efficiently in genome-scale networks as shown by our results, and it is furthermore applicable to graphs containing cycles. It is worth noting that substrate cycles allow an overall net conversion of cofactors but no net conversion of metabolic substrates (Gebauer et al., 2012), whereas the cycles found by the Kritz algorithm are graph-theoretical cycles that can exchange cofactors and substrates with other reactions. Due to the combinatorial nature of the network, the results can contain highly similar cycles that only differ by a few reactions. The same property is observed with EFMs, which led to the development of methods to classify EFMs based on common sets of reactions they contain in order to facilitate their biological interpretation (Pérès et al., 2011). We envisage that similar methods could be developed for cycles in the future.

We have unveiled cycles that play fundamental roles in adapting to changes in environmental conditions and explain known algal physiology. We were able to show a feedback loop existing in the presence of acetate, resulting in a decrease of glycolysis and an increase in or activation of the OPPP. These changes in carbon metabolism pathways all have implications on levels of ATP and NAPDH in the cell, which ultimately feed deeper into the respiratory pathways to ultimately reduce photosynthesis. Using cyclic decomposition analysis, we have unveiled novel theoretical cycles that could not be otherwise detected. This methodology provides a stronger and more comprehensive method to analyse the metabolism of any GEM, as we have shown using *C. reinhardtii* as an example.

## Author contributions

The project work was conducted by SC and MTS. The project was designed and supervised by MVK, JMS and GNJ. All authors have read and approved the final manuscript.
