# Supplementary material for: Cyclic decomposition explains a photosynthetic down regulation for Chlamydomonas reinhardtii
Source: Biosystems. 2017 Dec;162:119–27. doi: 10.1016/j.biosystems.2017.09.014 (PMC5720477; doi:10.1016/j.biosystems.2017.09.014)
Supplement: Supplementary file 4 [file mmc4.docx]

**Supplementary files**

Supplementary file 1: List of cofactors (Table S1), list of antiporter reactions (Table S2) and flux distributions calculated by pFBA (Table S3).

Supplementary file 2: List of cycles and associated flux in phototrophic condition (LNA)

Supplementary file 3: List of cycles and associated flux in mixotrophic condition (LWAC)
